# A Systematic Review of Household and Family Alcohol Use and Adolescent Behavioural Outcomes in Low- and Middle-Income Countries

**DOI:** 10.1007/s10578-020-01038-w

**Published:** 2020-08-12

**Authors:** Tahir Jokinen, Emma C. Alexander, Logan Manikam, Tausif Huq, Priyanka Patil, Darrin Benjumea, Ishani Das, Leslie L. Davidson

**Affiliations:** 1 GKT School of Medical Education, King’s College London, London, UK; 2 London North West University Healthcare NHS Trust, London, UK; 3 Aceso Global Health Consultants Limited, London, UK; 4 UCL Institute of Epidemiology and Health Care, University College London, London, WC1E 7HB UK; 5 Mailman School of Public Health, Columbia University, New York, USA

**Keywords:** Alcohol, Adolescent, Behaviour, Low- and middle-income countries

## Abstract

Exposure to alcohol misuse is considered an adverse childhood experience impacting on neurodevelopmental and behavioural outcomes in adolescents including substance use, mental illness, problem behaviours, suicidality, and teenage pregnancy. Most research on this issue has focussed on higher income countries, whereas patterns of alcohol use and related factors may be different in low- and middle-income countries (LMICs). This systematic review therefore seeks to collate all published studies from 1990–2020 on the topic set in LMICs. 43 studies were included, totalling 70,609 participants from 18 LMICs. Outcomes assessed included: substance use; depression/anxiety; suicidal ideation; problem behaviour; emotional dysfunction; teenage pregnancy; and self-harm. Despite heterogeneity in the studies identified, this review documented some association between exposure to household alcohol misuse and adverse adolescent outcomes in LMICs, including mental health problems, problem behaviours, and suicidality. The mechanisms leading to these outcomes are likely varied, and further research in different socio-economic and cultural contexts, particularly in the form of longitudinal studies, is called for.

**Electronic supplementary material:**

The online version of this article (10.1007/s10578-020-01038-w) contains supplementary material, which is available to authorized users.

## Introduction

Experiences in childhood and adolescence have been shown to have a significant impact on health in later life, primarily based on research from high income countries (HICs) [1, 2]. This might be through exposure to social and environmental factors directly leading to the development of particular diseases, or more indirectly, with childhood experiences shaping attitudes and future health behaviours. Some of these consequences become apparent relatively quickly, others develop much later in adult life. Of particular interest are those that become prominent during adolescence, as this has been identified as a stage when many enduring health problems (including mental and physical health as well as risky behaviours) first emerge [3, 4].

Adverse childhood experiences (ACEs), including parental or caregiver substance abuse, mental illness, conflict, neglect, or abuse, have been particularly associated with harmful adolescent neurodevelopmental and behavioural outcomes [5]. Children living in families where they are exposed to alcohol misuse by a caregiver have been described as having an increased likelihood of adverse experiences including abuse or neglect [6]. The negative association between maternal alcohol exposure in utero and development is well known; exposure can cause foetal alcohol spectrum disorders which include developmental and later mental health problems [7, 8], but the impact of indirect alcohol exposure via caregivers and negative adolescent outcomes has been comparatively less well described, particularly in low and middle income countries (LMICs). Harmful or problem drinking by parents, caregivers, or others in the household can disrupt family relationships, place adolescents under chronic stress, and lead to injury, abuse and neglect [1]. Associated consequences in terms of adolescent health can include suicidality, mental illness, substance use, teenage pregnancy, and a range of problem behaviours such as absence from school and internet addiction.

Comparatively little work has been done to investigate how household alcohol misuse impacts adolescents in the household in LMICs specifically. This demands attention, as patterns of alcohol use and misuse, household structure and dynamics, and childhood experiences vary considerably by country and culture [9]. Family units tend to be more cohesive in LMICs than in high-income countries, with large households and several generations under one roof, increasing the potential for exposure to alcohol misuse in the home from a large number of family members [10], especially in the presence of household crowding which is known to exacerbate mental health outcomes including alcohol abuse [11]. The multigenerational aspect could also conceivably be protective. Therefore, we aim to review and synthesise the results of studies from LMICs, in the hope that findings can inform policy recommendations and directions for future research.

Studies on childhood indirect exposure to alcohol misuse in the home have tended to report neurodevelopmental outcomes, whereas those on adolescents focus more on behavioural outcomes. Mechanisms through which associations might arise vary significantly between these two age groups and types of outcomes; therefore to maintain focus it makes sense for adolescents to be studied as an individual group [12]. The following review therefore reports specifically on adolescent behavioural outcomes, given that the relative balance between the rapid increase in the development of executive function and simultaneously an increase in impulsivity and independence may result in the opportunities for certain risky behaviours to occur [13]. A separate review completed in parallel to this one reports on the studies relevant to child neurodevelopmental outcomes [14].

Our aims are as follows:

1. To identify studies of the impact of excess alcohol consumption among household adults on adolescent developmental health outcomes (neurodevelopmental, cognitive and behavioural) in low- and middle-income countries and to evaluate the quality of the research
2. To explore whether the nature of alcohol use and misuse differs by individual family members (father, mother or other family member) in its impact on adolescent health outcomes (including neurodevelopment, cognitive and behavioural impact) in low- and middle-income countries

## Methods

A protocol for this review was published on the PROSPERO register in June 2017, registration number CRD42017070209.

### Eligibility Criteria

Studies were included in this review if they met the following criteria:

- Participants: individuals aged 10–18 years
- Exposure: household member engaging in alcohol misuse. ‘Household' included all relatives living in the household. 'Family' included parents and siblings
- Setting: low- and middle-income countries as defined by the World Bank [15]
- Outcome: Outcome measure of adverse adolescent behavioural and neurodevelopmental impacts (excludes outcomes directly related to alcohol exposure such as children’s own drinking behaviour as a result of adult alcohol exposure or in utero alcohol exposure)
- Language: Studies published in English, or with translation available
- Year: Published from 1990 or later

In order to be inclusive and avoid missing potentially relevant studies, a wide participant-eligible age range of 10–18 was used for this study [16]. Behavioural and neurodevelopmental changes of adolescence have been shown to begin with the onset of puberty, with hormonal changes associated with changes in neuronal development and cognitive function [17, 18]. Studies which only included a small minority of participants within this range were excluded. The lower limit of age 10 was selected in line with guidance from the WHO, who define adolescence as beginning at this age [19]. Age 10 is also the age at which most girls, who commence puberty prior to boys, have commenced puberty (defined by the onset of breast development) [20]. The upper limit of age 18 was selected as this is the legal age at which a subject is considered an adult in the majority of countries [21]. Studies which focused on antenatal, rather than household, alcohol exposure, were excluded due to confounding. Studies which described outcomes of abuse unconnected to characteristics of the child or adolescent were also not eligible.

The term ‘alcohol misuse’ i.e. harmful use (ICD-11 code F10.1) was understood as harmful in accordance with WHO guidelines, whether due to excessive volumes above recommended lower limits, or problematic patterns of drinking [22]. In order to ensure that no potentially relevant studies would be missed, the search term included ‘use’ to be deliberately broad and inclusive. Adolescents’ own use of alcohol was deliberately excluded as an outcome measure. This was partly because the relationship with household alcohol misuse has already been addressed in previous reviews [23, 24], and partly because this review sought to investigate more indirect impacts of alcohol misuse in the home. Adolescent drinking due to potential genetic elements, or due to easy availability of alcohol, was not of interest [25, 26].

### Information Sources

Five electronic databases were searched from 1990 to April 2020: Medline, EMBASE, OVID Global Health, Cochrane Library and PsychInfo. An original June 2017 search was updated in June 2018 and in April 2020.

### Search Strategy

The search strategy was structured as follows: “alcohol use” AND “household” AND “young person” AND “neurodevelopmental outcome(s)” with associated synonyms. Each category contained a range of terms. The full search strings used for MEDLINE are available in Supplementary File 1*.*

The results were then filtered using the Cochrane LMIC filter, modelled on World Bank definitions, to return studies set in low and middle income countries (LMICs) [27]. This filter has been previously used in systematic reviews of similar nature [28–31]. During targeted abstract and full text review, further screening made use of the up to date World Bank list of LMICs [15].

### Study Selection and Data Extraction

After the studies were downloaded, and the Cochrane LMIC filter had been applied, each title and abstract was reviewed by one reviewer and uncertainties checked by a second against the inclusion criteria. Shortlisted full-text articles were subsequently checked by two separate reviewers. This led to the population of the final list of studies to be included.

A standardised pre-piloted extraction form was developed, tested on 10 articles and revised iteratively as needed. Extracted information included:

1. Study characteristics: setting, study design, method of data-analysis;
2. Participants: study population, number of participants in each group, patient characteristics
3. Child or adolescent health outcome (as reflected in primary outcome)
4. Household adult alcohol exposure or definition (as reflected in secondary outcome)
5. Household location, income, food insecurity, asset index and family factors and other factors (if available)

Each study type was classified e.g. cohort, cross-sectional study, according to standard definitions [32]. Each study was also classified as relevant to adolescents, children, or both.

### Results Synthesis

The evidence reviewed is presented as a narrative report due to the wide range of methodologies used, populations, and outcomes. It was an objective of this study to incorporate any reported behavioural outcomes. Consequently, there were no pre-specified outcome measures; rather, these were identified and categorised iteratively by the research team according to the outcomes reported in the eligible studies. The results for adolescents covered the following behavioural and mental health outcomes:

- Substance use (other than alcohol)
- Depression/anxiety and other psychiatric disorders
- Emotional dysfunction
- Problem behaviour
- Suicidal ideation and behaviour
- Teenage pregnancy
- Self-harm (non-suicidal)

### Quality Assurance

In our review, given the predominance of observational studies, the National Heart, Lung and Blood Institute (NHLBI) Quality Assessment Tool for Observational Cohort and Cross-Sectional Studies was utilised, or the equivalent tool for Case–Control studies if applicable [33]. The former tool asks 14 questions with answers of ‘Yes’, ‘No’ or ‘Other’, such as ‘*Was the exposure(s) assessed more than once over time?’* Two reviewers independently screened each study shortlisted for inclusion and awarded one point for each ‘yes’ answer, with additional arbitration by other team members where required, in order to reach an overall score. A maximum score of 14 (12 for Case–Control studies) was available for each paper, and a minimum score of 0, with higher scores indicating a high-quality paper relevant to our objectives. The scores were then used to produce an overall rating of ‘Good’, ‘Fair’ or ‘Poor’ relevant to the review, with studies rated as ‘Poor’ excluded from inclusion in the main results section.

## Results

**Fig. 1:**
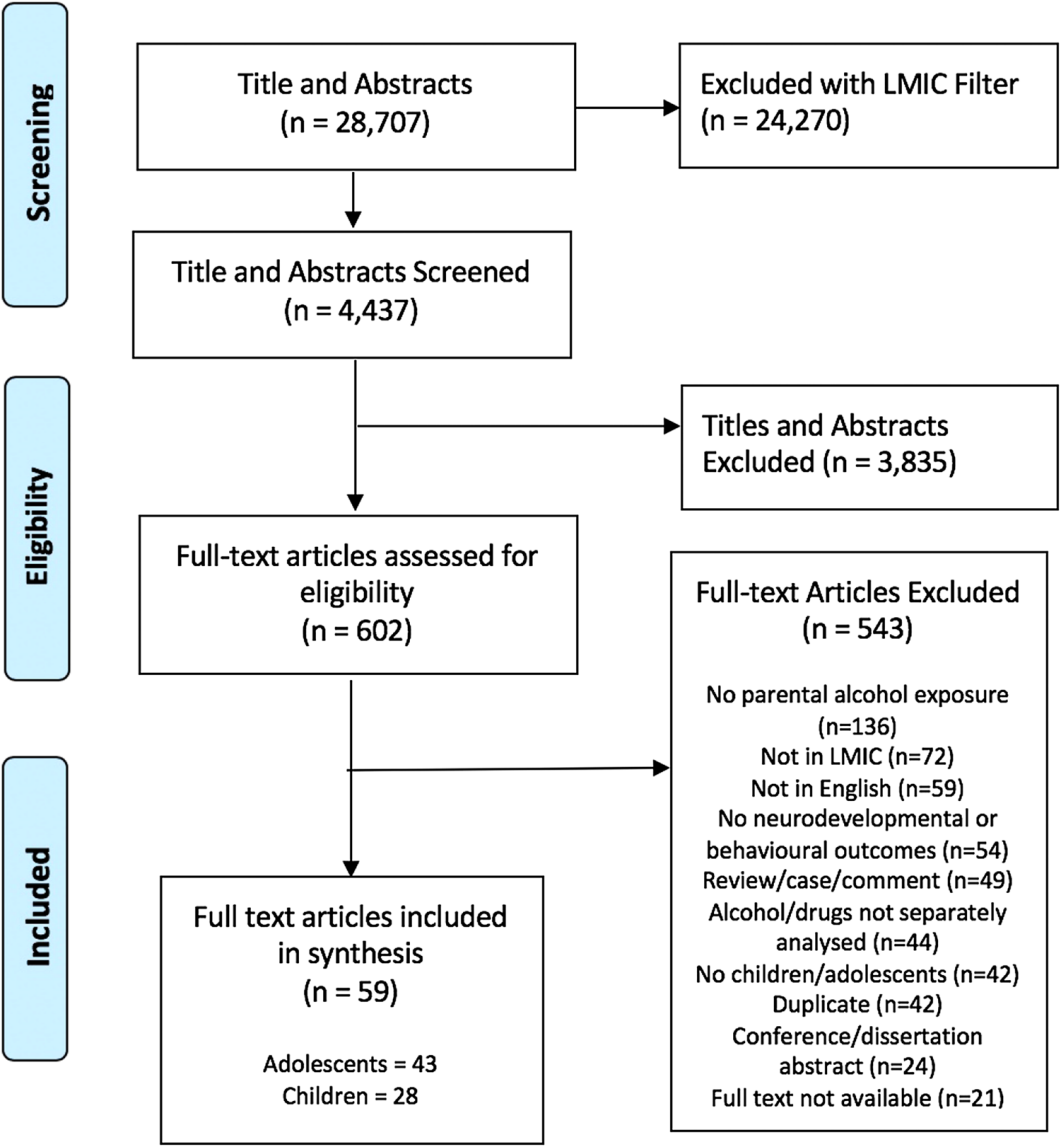
Study selection process

In total, 28,707 titles and abstracts were downloaded from the chosen databases. After filtering with the Cochrane LMIC filter [27], 4437 results were screened by title and abstract. 602 papers were subsequently selected for full text review. The process of study selection is illustrated in Fig. 1.

### Study Characteristics

In total, 43 studies were included in this review of adolescents: 37 cross-sectional studies, 3 case–control studies, 2 cohort studies, and 1 mixed-methods study. Across the studies, 70,609 participants were included, from 18 different lower- and middle-income countries. The countries of origin of the included studies are reported in Table 1. Key characteristics are described in Table 2.

**Table 1:** Countries of origin of included studies

Country	Number of studies
India	9 studies [36, 43, 48, 58–60, 64, 68, 74]
Turkey	9 studies [39, 51–54, 56, 57, 67, 71]
Brazil	6 studies [40, 46, 47, 49, 75, 85]
South Africa	3 studies [35, 55, 66]
Kenya	2 studies [65, 73]
Rwanda	2 studies [37, 42]
Uganda	2 studies [69, 72]
Thailand	2 studies [41, 44]
Mexico	1 study [34]
China	1 study [70]
Ghana	1 study [38]
Sri Lanka	1 study [62]
Ukraine	1 study [45]
Botswana	1 study [50]
Morocco	1 study [63]
Multi-country (Laos, Mongolia, Nepal, Sri Lanka)	1 study [61]

**Table 2:** Summary of included studies

Author	Country	Study type	Sample size	Population	Age range	Household alcohol exposure	Household location	Adolescent outcomes assessed	NHLBI Quality score	NHLBI Quality overall assessment
Andrade [46]	Brazil	Cross-sectional	1231	Public school students	14–17	Family	Urban	Substance use	10	Good
Betancourt [42]	Rwanda	Cross-sectional (as part of larger Case–control study)	680	Young people affected by and living with HIV	11–17	Family	Rural	Suicidal ideation/behaviour	6	Fair
Burlaka 2017 [45]	Ukraine	Cross-sectional	251	Mothers and children	9–16	Mother	Mixed	Emotional dysfunction	9	Good
Caputo and Bordin [40]	Brazil	Case–control	408	Sexually active teenage girls	13–17	Family	Urban	Teenage pregnancy	9	Good
Catani [62]	Sri Lanka	Cross-sectional	296	Young people attending school	9–15	Parents	Urban	Depression/anxiety and other psychiatric disorders	7	Fair
Chaudhury [37]	Rwanda	Mixed methods	93	Families affected by caregiver HIV	7–17	Caregiver	Rural	Depression/anxiety and other psychiatric disorders	11	Good
Ciftci Demirci [56]	Turkey	Cross-sectional	1969	Young people seeking treatment for substance use disorder	11–20	Parents	Urban	Substance use	8	Fair
da Rocha [85]	Brazil	Case–control study	146	Those with an IQ less than 70 referred to a special education institution	7–19	Relative/family	Urban	Cognitive delay/disorder	6	Fair
Evren [54]	Turkey	Cross-sectional	4957	10th grade students in Istanbul	Mean 16.54 (SD 2.72)	Parents	Urban	Substance use	7	Fair
Faler [75]	Brazil	Case–control	431	Adolescent females	14–16	Parents	Urban	Teenage pregnancy	8	Fair
Francisco [34]	Mexico	Cross-sectional	785	Pregnant adolescents	13–19	Parents	Urban	Substance use; teenage pregnancy	9	Good
Gulati and Dutta [43]	India	Cross-sectional	245	Adolescents from families living in conditions of poverty	12–16	Parents	Rural	Emotional dysfunction	8	Fair
Guvendeger Doksat [71]	Turkey	Cross-sectional	2518	Young people admitted to psychiatric hospital	Mean 16*	Family	Urban	Suicidal ideation/behaviour; self-harm	8	Fair
Hamdulay and Mash [55]	South Africa	Cross-sectional	438	Students attending grade 8 and 11 classes	14–17	Household	Urban	Substance use	7	Fair
Jaisoorya 2018 [48]	India	Cross-sectional	7560	School Students	12–19	Family	Mixed	Emotional dysfunction	10	Good
Jogdand and Naik [68]	India	Cross-sectional	600	General	6–18	Caregiver	Urban	Problem behaviours	7	Fair
Karatay and Bas [67]	Turkey	Cross-sectional	613	High school students	Approx 14–18 (9th–12th grade)	Family	Urban	Emotional dysfunction	8	Fair
Khasakhala [73]	Kenya	Cross-sectional	250	Youth attending clinic with psychiatric or substance use disorder	13–25	Father	Urban	Suicidal ideation/behaviour	7	Fair
Khasakhala [65]	Kenya	Cross-sectional	250	Youth attending psychiatric clinic	13–22	Father	Urban	Depression/anxiety and other psychiatric disorders	7	Fair
Kheokao [41]	Thailand	Cross-sectional	5184	School students	9–22	Family	Not specified	Problem behaviours; cognitive delay/disorder	8	Fair
Lee 2019 [61]	Laos, Mongolia, Nepal, Sri Lanka	Cross-sectional	4098	High school students	Approx 12–17 (grade 7–11)	Parents	Urban	Depression/anxiety and other psychiatric disorders	8	Fair
Madruga [47]	Brazil	Cross-sectional	761	Secondary school children	14–19	Household	Mixed	Substance use	8	Fair
Mansharamani [58]	India	Cross-sectional	100	Outpatients and inpatients at a psychiatric ward of a tertiary facility	4–14	Parents	Mixed	Cognitive delay/disorder; depression/anxiety and other psychiatric disorders; emotional dysfunction	6	Fair
Meyer [44]	Thailand	Cross-sectional	23	Children/youth	9–17	Caregiver	Refugee camp	Problem behaviours; emotional dysfunction	6	Fair
Omkarappa and Rentala [59]	India	Cross-sectional	200	Secondary school students at government schools	12–16	Parents	Urban	Depression/anxiety and other psychiatric disorders; emotional dysfunction	9	Good
Ozcan and Ozcan [53]	Turkey	Cross-sectional	4767	Middle school and high school students	10–18	Parents	Urban	Substance use	8	Fair
Pillay and van der Veen [66]	South Africa	Cross-sectional	100	Children admitted to an inpatient psychiatric unit	4–17	Parents	Urban	Depression/anxiety and other psychiatric disorders	6	Fair
Praharaj [36]	India	Cross-sectional	9	Street children and adolescents abusing typewriter correction fluid	10–17	Father	Urban	Substance use	5	Poor
Rahi [60]	India	Cross-sectional	620	Slum children	4–14	Father	Urban	Depression/anxiety and other psychiatric disorders	7	Fair
Riva [50]	Botswana	Cross-sectional	1936	Students in public secondary schools	IQR 14–17	Household	Urban	Substance use	9	Good
Singh [74]	India	Cross-sectional	94	Adolescents admitted after attempted suicide by ingestion	15–18	Parents	Urban	Suicidal ideation/behaviour	6	Fair
Singh [64]	India	Cross-sectional	542	Secondary school students	14–18	Father	Urban	Depression/anxiety and other psychiatric disorders	7	Fair
Sonmez [51]	Turkey	Cross-sectional	8483	High school students	Mean 16.12 (SD 0.99)	Parents	Urban	Substance use	6	Fair
Swahn [69]	Uganda	Cross-sectional	457	Youth living in the slums of Kampala	14–24	Parents	Urban	Problem behaviours	9	Good
Swahn [72]	Uganda	Cross-sectional	457	Youth living in the slums of Kampala	14–24	Parents	Urban	Suicidal ideation/behaviour	9	Good
Tavares [49]	Brazil	Cross-sectional	2410	Adolescents 9-11^th^ grades	10–19	Family	Urban	Substance use	8	Fair
Tot [52]	Turkey	Cross-sectional	3282	Sixth grade, tenth grade and university students	Mean 15.75*	Parents	Urban	Substance use	7	Fair
Wood [35]	South Africa	Cross-sectional	20	Children and adolescents convicted of sex offences	7–15	Parents/household	Mixed	Problem behaviours	6	Fair
Xing [70]	China	Cross-sectional	12,470	High school students	11–19	Family	Mixed	Suicidal ideation/behaviour	8	Fair
Yarney [38]	Ghana	Cohort	17	Young people orphaned by AIDS	12–17	Caregiver	Mixed	Problem behaviours	6	Fair
Yurtseven [57]	Turkey	Cohort	131	Adolescents presenting to ED with drug use and positive tests	13–18	Parents	Mixed	Substance use	7	Fair
Zeren [39]	Turkey	Cross-sectional	351	Juveniles involved in a crime as suspects or victims	0–18 (majority 16–18)	Parents	Urban	Problem behaviours	7	Fair
Zouini [63]	Morocco	Cross-sectional	375	High school students	15–18	Parents	Urban	Problem behaviours, Depression/anxiety and other psychiatric disorders; emotional problems	6	Fair

*Overall population SD not reported

### Sample and Household Characteristics

Four studies included only female adolescents, of which one looked only at pregnant females [34]; two at only males [35, 36], and the others included both genders. Most studies recruited adolescent participants from schools, although eight studies recruited directly from psychiatric facilities, both inpatient and outpatient. One study looked specifically at alcohol misuse impact on adolescents affected by caregiver HIV [37], one study looked at adolescents orphaned due to AIDS [38]. Another examined young sex offenders [35], and another juveniles who committed or were victims of crimes [39].

The majority (30 studies) were of urban households (e.g. [40]). One did not specify whether urban or rural [41]. Eight studies included households from mixed locations, incorporating both urban centres and rural areas (e.g. [35]). Three were rural locations [37, 42, 43], and one study was in a refugee camp [44].

### Quality of Included Studies

NHLBI Quality Assessment Tool included studies ranged between 5 and 11 (out of 14). These scores were used to produce an ‘overall rating’ for which 32 studies were rated as ‘fair’, ten were rated ‘good’ and one ‘poor’. Overall ratings can be seen in Table 2. Studies commonly scored well for subjects being recruited from appropriate similar populations, and for clearly defining outcome measures. Few assessed exposures stratified by different levels of exposure (i.e. different levels of alcohol exposure).

Seventeen of the studies adjusted statistically for confounding variables. Only five studies measured different levels of exposure to alcohol; the remainder did not differentiate. All but one of the studies were of sufficient quality to be included in the main body of the results [36].

### Household Alcohol Consumption

Alcohol exposure is summarised in Table 3. 'Family' included parents and siblings. 'Household' included all relatives living in the household. A number of studies that looked at parental alcohol exposure reported results for maternal and paternal exposure separately. One study looked at the effect of maternal alcohol consumption alone.

**Table 3:** Summary of household alcohol exposure sources

Household alcohol exposure	Number of studies	Adolescent Outcomes
Parents	19 [34, 39, 43, 61–63, 54, 56–59, 61, 51–54, 56–58]	Depression/anxiety and other psychiatric disorders; substance use; teenage pregnancy; problem behaviours; emotional dysfunction; cognitive delay/disorder; suicidal ideation/behaviour
Family	11 [35, 40–42, 46, 48, 49, 67, 70, 71, 85]	Suicidal ideation and behaviour; teenage pregnancy; self-harm; substance use; problem behaviours; emotional dysfunction; cognitive delay/disorder
Caregiver	4 [37, 38, 44, 68]	Depression/anxiety and other psychiatric disorders; problem behaviours; emotional dysfunction
Household	4 [35, 47, 50, 55]	Substance use; problem behaviours
Father	4 [60, 64, 65, 73]	Suicidal ideation and behaviour; depression/anxiety and other psychiatric disorders
Mother	1 [45]	Emotional dysfunction

Few of the studies made any attempt to quantify alcohol use or to consider the different forms in which it was consumed; as an example, Burlaka et al. estimated the number of annual drinks [45]. Among others, Andrade et al. set a minimum threshold for being defined as a ‘high consumer’ of 250 ml of beer or 40 ml of distilled beverages on more than 1–2 days a week [46]. Many studies referred to ‘alcohol use’, ‘alcohol abuse’, ‘alcoholic’, and ‘problem drinking’, however definitions of these categories were often vague or non-existent. Four recognised alcohol scales were described across the studies: AUDIT [37], an Alcohol Abuse checklist [43], the Hispanic Americans Baseline Alcohol Survey [47], and the WHO-Thai Health Harm to Others from Drinking questionnaire [48].

### Behavioural Outcomes

Behavioural outcomes assessed by included studies are summarised in Table 4. Some studies included more than one type of outcome. Results were heterogeneous, and therefore key study findings have been described individually in the sections below.

**Table 4:** Summary of adolescent outcomes in included studies

Adolescent outcome	Number of studies
Substance use (other than alcohol)	12 [34, 46, 47, 49, 50, 49–55]
Depression/anxiety and other psychiatric disorders	10 [37, 58–66]
Emotional dysfunction	8 [43–45, 48, 58, 59, 63, 67]
Problem behaviour	8 [35, 38, 39, 41, 44, 63, 68, 69]
Suicidal ideation and behaviour	6 [37, 42, 70, 72–74]
Teenage pregnancy	3 [34, 40, 75]
Cognitive delay/disorder	3 [41, 58, 85]
Self-harm (non-suicidal)	1 [71]

#### Substance Use (Other Than Alcohol)

The most frequently linked adolescent outcome was substance use (of substances other than alcohol). Twelve studies showed some association between household alcohol misuse and adolescent substance use; tobacco was the most common outcome studied, but other substances were also mentioned. As stated in the methods, adolescents’ own use of alcohol was not a focus of this review. In Tavares et al. (Brazil), the prevalence ratio of drug use was 1.50 (95% Confidence Interval (CI) 1.19–1.90, p = 0.000) in adolescents who had alcoholism in the family [49]. This was similar to Riva et al. (Botswana) who reported a relative risk of 1.7 (95% CI 1.4–2.1) of illicit drug use in those with a problem drinker at home; however, this factor was no longer significant after incorporation in multivariable models [50]. In Madruga et al. (Brazil), the odds ratio for any illegal drug use in those exposed to domestic violence related to alcohol was 5.29 (95% CI 1.52–18.38) [47]. Sonmez et al. (Turkey) investigated impact of mothers and fathers drinking separately [51]. They found a significantly greater proportion of adolescents whose mothers misused alcohol used a number of substances compared to adolescents whose mothers did not drink. These included tobacco (38.2% vs 20.9%), cannabis (3.9% vs 1.0%), cocaine (1.3% vs 0.1%), heroin (0.9% vs 0.1%), and benzodiazepines (3.4% vs 0.2%) (p < 0.01 for all) [51]. Similar positive associations were found for paternal alcohol use (p < 0.05 for all, except adolescent heroin use, which was not statistically significant).

Regarding smoking, Tot et al. (Turkey) reported similar results for the prevalence of adolescent tobacco use; 31.7% in the children of maternal alcohol drinkers compared to 23.5% in children of maternal non-drinkers (p = 0.001); the equivalent figure for children whose fathers were alcohol drinkers being 28.0% vs 20.9% (p = 0.0001) [52]. Ozcan and Ozcan (Turkey) found with binomial logistic regression that paternal use of alcohol increased adolescent probability of smoking by 1.48 (p = 0.019), but maternal use had no significant effect [53]. Andrade et al. (Brazil) found a statistically significant association between adolescent tobacco smoking and maternal alcohol use (Adjusted Odds Ratio (OR) 1.91 (95% CI 1.00–3.66), p = 0.049); however, for paternal alcohol use there was no significant association (Adjusted OR 2.37 (95% CI 0.86–6.47) [46]. Evren et al. (Turkey) identified an increased risk of adolescent tobacco use when participants experienced problems due to the alcohol use of parents (OR 1.62, 95% CI 1.31–1.99) [54]. However, Francisco et al. (Mexico) in a study of adolescent girls with planned or unplanned pregnancy reported that alcohol drinking in the home was not associated with smoking in either group [34].

Hamdulay and Mash (South Africa) found that 59.8% of adolescents exposed to alcohol consumption at home smoked cannabis, compared to 40.2% among those who were not exposed (p = 0.05) [55]. Ciftci Demirci et al. (Turkey) found that 9.3% of adolescents admitted for treatment of substance use disorder (no control group) had parents who heavily abused alcohol; in Yurtseven et al., also in Turkey, 83% of illicit-drug using adolescents had a parent who consumed alcohol more than once a month [56, 57].

#### Depression/Anxiety and Other Psychiatric Disorders

Ten studies explored depression and other psychiatric disorders associated with exposure to household alcohol, with mixed results. Two studies in India reported significantly greater mean scores for depression and anxiety in the children of alcoholics compared to the children of non-alcoholics [58, 59]. In particular, Mansharamani et al. also found a significant difference in total scores using the ‘Childhood Psychopathology Measurement Schedule’ which was mean 6.10 in children of alcoholics, and mean 3.12 in children of non-alcoholics (p = 0.0001) [58]. Chaudhury et al. (Rwanda) noticed adolescents' self-reported anxiety and depression improved following an intervention aimed at reduction in caregiver alcohol use; the association reaching statistical significance [37]. In a sample of 620, Rahi et al. observed a higher prevalence of psychopathological disorder in children whose fathers abused alcohol than those whose fathers did not (20.2% vs 13.6%, p < 0.05) [60]. In a multi-country study in Laos, Mongolia, Nepal, and Sri Lanka, Lee et al. reported significantly increased odds ratios of psychological distress in the children of parents who used alcohol across three of four countries in unadjusted analyses; however this association was no longer significant in multivariate analyses [61]. Catani et al. found that paternal alcohol consumption was associated with increased family violence (p < 0.01) in a study in Sri Lanka [62]. In this study, family violence in turn was associated with significantly increased risk of PTSD in adolescents (p < 0.001), though there was no direct examination of the association between parental alcohol consumption and PTSD [62].

The other four studies investigating depression and other psychiatric disorders reported no direct association between household alcohol exposure and depression and anxiety disorders. In Zouini et al. there was no significant difference in mean scores for depression, somatisation, obsessive compulsiveness, psychoticism and anxiety between adolescents reporting parental alcohol us and the comparison group [63]. When assessing the prevalence of depression associated with the frequency of paternal drinking, Singh et al. (India) found that of those whose fathers drank every day (n = 21), 67% had depression; of those whose fathers drank occasionally (n = 100), 50.0% had depression; and of those whose fathers drank rarely (n = 54), 57.4% were depressed [64]. Khasakhala et al. (Kenya) found that there was no statistically significant association between major depressive disorder in adolescents whose fathers drink any alcohol, compared to adolescents whose fathers did not (OR 1.13, 95% CI 0.65–1.97) [65], and Pillay and van der Veen found there was no statistically significant association between prior substance abuse at home, 93% involving alcohol, and depression amongst child and adolescent psychiatric inpatients, in a small sample in South Africa [66].

#### Emotional Dysfunction

Eight studies explored varied emotional effects on adolescents exposed to household alcohol misuse. For example, Omkarappa and Rentala (2019) (India) found that children of alcoholics had a mean self-esteem score lower than children of non-alcoholics (19.54 vs 26.46, p = 0.001). Meyer et al., in a qualitative study of adolescents in a refugee camp in Thailand, described parental/caregiver drinking leading to fighting between caregivers and adolescents feeling ‘afraid’, ‘shy’, ‘mentally affected’, ‘melancholy’, and ‘stressed’ [44]. It was suggested that parents’ drinking and fighting were chronic stressors. In a study in India of 7560 students, 53.9% of boys and 33.0% of girls reported experiencing psychological harm from others’ drinking, with examples including being called names or insulted [48].

However, five studies reported mixed/negative outcomes in this domain. Karatay and Bas (Turkey) initially found that self-efficacy score was lower in those who had alcohol users in the family vs those who did not (88.21 vs 95.81, p < 0.001) but this was no longer significant after multiple regression analysis [67]. Gulati and Dutta (India) found in a multiple linear regression analysis that the father’s alcohol abuse status was not significant as a risk variable for internalised or externalised behaviours [43]. Similarly, Burlaka et al. (Ukraine) found no association between internalising behaviours and maternal alcohol misuse, and Mansharamani et al. found no difference in scores for ‘physical illness with emotional problems’ between children with alcoholics and children of non-alcoholics (mean 0.48 vs 0.46, p = 0.88) [45]. Finally, Zouini et al. (Morocco) found no significant difference in mean scores for interpersonal sensitivity between the adolescents reporting parental alcohol use and the comparison group [63].

#### Problem Behaviour

Eight studies investigated a range of problem behaviours associated with exposure to household alcohol, with mixed associations found. Jogdand and Naik (2014) (India) found 56.3% of adolescents with parent/carer alcoholism exhibited behavioural problems (OR 1.56, 95% CI 1.12–2.17) [68]. Meyer et al., in a qualitative study of adolescents in refugee camps in Thailand, reported behavioural effects of exposure to adult drinking: adolescents 'don't attend school, don't go among people, go against parents, hiding’ [44]. Kheokao et al. (Thailand) reported a significant correlation between family drinking and absenteeism [41]. Yarney et al., in another qualitative study looking at adolescents in Ghana orphaned by AIDS, suggested that orphans were vulnerable to “pilfering and other social vices” when caregivers spent money on time on social activities including drinking, suggesting this was because it reduced worsened finances and caregiver presence at home [38]. In a study of 20 young people convicted of sex offences in South Africa, Wood et al. found that 75% of the sample one or more family members abused alcohol [35]. However, in Swahn et al. (Uganda) an initial elevated odds ratio (OR 4.59, 95% CI 1.18–17.96) of violence perpetration in youth who reported parental neglect due to alcohol use was no longer significant after adjustment (Adjusted OR 2.55 (95% CI 0.48–13.63) [69]. In Zeren et al., when comparing characteristics of young people suspected of committing, or victims of, crimes, there was little difference between the proportion of suspects and victims with fathers who abused alcohol (4.9% vs 5.6%); this was not assessed for statistical significance [39]. Only 3 participants (1%) had mothers who abused alcohol. In Zouini et al. (Morocco) there was no significant difference in mean scores for hostility in those adolescents reporting parental alcohol use problems versus the comparison group [63].

#### Suicidal Ideation and Behaviour

Six studies examined the associations, varying in strength, between exposure to household alcohol and suicidal ideation and/or behaviour. Xing et al. found an increased prevalence of suicide attempts in the past year in those who had a family member with an alcohol abuse problem (4.4% vs 2.2%, p < 0.001) [70]. Guvendeger Doksat et al. (Turkey) found that a history of alcohol use by parents increased the risk of suicide attempts in their adolescents hospitalised for substance use (OR 1.664, p = 0.001) [71]. Swahn et al. (Uganda) found that of youth reporting parental neglect due to alcohol use, there was an elevated adjusted odds ratio for suicidal ideation (Adjusted OR 2.09 (1.16–3.77) [72]. Conversely, Khasakhala et al. (Kenya) found that there was no significant association between the presence of paternal alcohol use disorder and the occurrence of suicidal behaviour (OR 1.86, 95% CI 0.88–3.92) [73]. There were two descriptive studies; Betancourt et al., in a study in rural Rwanda, found that 4 out of 20 youths reporting current suicidality identified alcohol abuse in the family as a reason for suicidal behaviour (42). In Singh et al. (India), 9.6% of adolescents admitted to a paediatric unit for suicide attempts by ingestion had alcoholism in the family [74].

#### Teenage Pregnancy

Three studies suggested an association between teenage pregnancy and household alcohol misuse. Caputo and Bordin (Brazil) found a statistically significant association, with 17% of pregnant girls having a family member getting drunk more than once a week compared to 8.4% of non-pregnant girls (OR 2.2, 95% CI 1.1–4.3, p = 0.014) [40]. It was suggested that harmful alcohol use in the family acted as a permanent stress factor. Faler et al. (Brazil) also found an association (OR 1.33, 95% CI 1.05–1.68, p = 0.016) with parental alcohol problems [75]. Francisco et al. (Mexico) looked specifically at pregnant girls to see whether pregnancy was planned or unplanned, finding that alcohol consumption at home was present in 60.4% of planned pregnancies compared with 77.5% of unplanned pregnancies (p < 0.05) [34].

#### Self-harm

Guvendeger Doksat et al. found that in a large study of adolescents admitted to a psychiatric hospital in Turkey for treatment of substance use disorder, non-suicidal self-injury was present in over half of those whose relatives used alcohol compared to those who did not (57.3% vs 42.7%, p = 0.016) [71]. The association between paternal alcohol use and the presence of non-suicidal self-injury was statistically significant (63.9% vs 36.1%, p < 0.0001), but not the presence of maternal alcohol use (68.8% vs 31.2%, p = 0.175), although the sample size was small in the latter case.

### Neurodevelopmental Outcomes in Adolescents

Although this systematic review sought to include neurodevelopmental outcomes (such as attention deficit hyperactivity disorder, autism spectrum disorder, global developmental delay, intellectual disability), the majority of studies of adolescents identified did not investigate these outcomes, and no studies investigated these outcomes in a purely adolescent population. The parallel paper to this review, focusing on neurodevelopmental and behavioural outcomes in children, reported a high number of relevant papers, indicating a greater focus of the research field into specifically neurodevelopmental outcomes at the younger years.

### Mechanisms/Subgroup Analysis

Very few studies provided information on household socioeconomic status, malnutrition, or stunting. Where such information was provided, it was not linked to alcohol consumption or behavioural outcomes. Therefore, no conclusions can be drawn about the impact of these factors on the associations found.

The majority of studies focused on households in urban locations. Trends in terms of outcomes were similar across studies in urban and mixed locations. Due to the variety of outcomes and the lack of studies of rural households, it is not possible to comment on differences between urban/rural households, except that no outcomes were reported as unique to rural households.

The role of adolescent resilience as a mediating factor was raised by a few studies; adolescents with higher levels of resilience were reported as being better able to withstand the impact of household alcohol misuse, exhibiting less emotional dysfunction and problem behaviours. Other studies in high income countries have noted similar findings [37].

## Discussion

### What is Already Known on This Topic

Exposure to excessive household alcohol drinking is known to increase the risk of adverse child and adolescent health outcomes in high income countries [76]. This is known to be particularly true for maternal alcohol consumption [77]. These adverse outcomes are known to include a wide range of behavioural problems, cognitive effects, and increased rates of mental illness, as well as physical health problems [1]. Isolated studies have suggested the same relationship between adverse experiences and outcomes is true in low- and middle-income countries (see, for example, [78, 79]).

### What This Study Adds

This review confirms that, despite the heterogeneity of results, exposure to more than modest household alcohol use seems to be associated with some negative adolescent behavioural outcomes in many lower- and middle-income countries, just as in higher income countries. Many studies found statistically significant associations between household alcohol misuse and adolescent mental illness, problem behaviours, self-harm, and teenage pregnancy. Both maternal and paternal alcohol misuse emerged as key risk factors contributing to impact on adolescents.

Studies included in this review also speculated on mechanisms that might explain the link between household alcohol misuse and adverse adolescent outcomes. Parental alcohol misuse was theorised to act as chronic stressor, a marker of broader family dysfunction, and a shaper of adolescent attitudes to lifestyle and health behaviours. Studies frequently did not adjust for confounding factors, so it is not possible to identify a direct causal link between household alcohol alone and adverse impacts.

The heterogeneity in the results, the relative lack of statistical approaches and of appropriate control groups, necessitate that more studies be undertaken, using control groups or population samples, with clear definitions of use and misuse of alcohol and/or careful quantification of household alcohol use, in order that more concrete conclusions can be drawn. Alcohol use patterns including amount, frequency, type, and location of drinking need to be measured. Studies need to include measures of other factors which could increase adolescent risk such as socioeconomic deprivation, education, peer influence, and protective factors within the family and household.

Control groups were lacking in several studies; prospective cohort studies would address this. The inclusion of questions on household alcohol use and misuse in future prospective cohort studies of adolescents would provide valuable data. Numerous high-income countries already conduct regular surveys of adolescent health; questions on household alcohol misuse in all countries could be added to these [80, 81]. Better designed case control studies would be recommended, using as cases adolescents experiencing an outcome identified in this review and securing better information on both maternal, paternal and household alcohol use. As most of the studies thus far have focused on urban settings, more research in rural households would add to generalisability. Risk factors, negative adolescent outcomes, and potential mechanisms may well be significantly different in rural environments. Similarly, there is a need for studies to address other LMICs not included in this review, results from which would add nuance, highlight differences, and expand on our findings.

Further research also needs to be done to illuminate the mechanisms through which household alcohol misuse may impact on negative behavioural outcomes among adolescents. The absence of robust data on other exposures and the lack of comparison groups necessitates further research to allow evaluation of speculated mechanisms. Inclusion of information on important covariates, household income, partner violence, harsh discipline, physical or sexual adolescent abuse, and family functioning would allow research to consider confounding or mediating factors and interaction affecting the relationship between household alcohol misuse and negative adolescent outcomes. Previous studies have included alcohol as one of a list of ACEs [1] whereby it has not been possible to explore the independent role of household alcohol misuse and its relationship with other adverse experiences.

Significantly, very few of the included studies attempted to explicitly explore the relationship between amount of alcohol consumed and adolescent outcomes. Loose definitions of 'alcohol abuse' or 'problem drinking' were used to compare outcomes with little quantification of amount or context. It is not at all clear from the studies available whether there is a correlation between amount of alcohol exposure and severity of or frequency of adolescent problems. Consequently, it has not been possible to identify whether or not there is a level of household alcohol use that might be considered safe, or indeed to suggest what amount might be harmful. The nature of exposure to household alcohol was also poorly defined in the included studies. There was little indication of the extent to which adolescents were directly exposed either to drinking or drunkenness; it was not clear whether alcohol use mostly occurred at home in the adolescents' presence or outside the house. Furthermore, it was not always clear whether exposure to alcohol was historic or contemporaneous. This distinction may have important consequences in terms of outcomes, as well as potentially influencing causal relationships and consequently policy development. The majority of studies took place in urban areas, indicating a large proportion of children in LMICs have thus far been excluded from research in this area. Finally, though the systematic review also aimed to summarise adolescent studies describing neurodevelopmental outcomes, no study focused on this outcome in this age range.

### Limitations of this Review

Limitations of the review included the decision to exclude studies published before 1990, which meant that potentially relevant studies may have been missed. Excluding studies not available in English meant that some potentially important studies could not be considered. As in any systematic review, publication bias means that studies that did not find associations tended to be underrepresented.

The decision to include a wide age range of subjects from 10 to 18 meant that it was possible that a small minority of subjects had not yet started puberty, or had completed puberty, and were therefore not ‘adolescents’ in the traditional sense. This was done in order to avoid excluding otherwise relevant high quality studies. Detailed subgroup analyses of these studies would not have been possible, as the majority merely reported ages and did not assess puberty status, or report results by age categories. Definitive assessment of puberty and adolescence is difficult, a subject of ongoing academic debate, and can be based on variable categories and guidelines [82, 83]. Age is a common proxy for adolescence, and puberty is complex to assess (see RCPCH guidelines [84]). It is inevitable therefore that a small amount of misclassification may have occurred.

This review incorporated studies from a wide range of LMICs across Asia, Africa, and South America. Findings may well be somewhat generalisable. This being said, LMICs are not homogeneous, and varying cultural and socio-economic circumstances directly impact both understandings of alcohol misuse and adolescent outcomes, thereby limiting transferability of findings. In particular, given that only English language studies were included, otherwise relevant research may have been overlooked. Given that attitudes towards alcohol use and misuse, and particularly understandings of what is excessive or inappropriate, vary widely across cultural contexts, conclusions should be extended to different contexts with caution.

## Summary

This review shows that exposure to household alcohol misuse in the context of low- and middle-income countries is associated with a range of adverse adolescent outcomes. This was the case across a wide range of countries. Although results were heterogeneous and amounts of alcohol poorly quantified, some statistically significant associations were described between parental alcohol misuse and adolescent suicidality, depression, anxiety, substance use, problem behaviour, teenage pregnancy, and self-harm. Further research is called for, with more studies needed to allow hypotheses to be tested, and study of differences associated with country-specific contexts. In particular, careful quantification of alcohol misuse and characterization as risky drinking, dependent drinking or binge drinking might help to better establish the impact of exposure on the adolescents in the home. In addition, inclusion of key covariates and study designs including control groups would help an investigation of the mechanisms by which alcohol exposure is associated with these outcomes.

## Electronic supplementary material

Below is the link to the electronic supplementary material.

Supplementary file1 (DOCX 14 kb)
