# Supplementary material for: A Systematic Review of Household and Family Alcohol Use and Adolescent Behavioural Outcomes in Low- and Middle-Income Countries
Source: Child Psychiatry Hum Dev. 2020 Aug 12;52(4):554–70. doi: 10.1007/s10578-020-01038-w (PMC8238760; doi:10.1007/s10578-020-01038-w)
Supplement: Supplementary file 1 — Supplementary file1 (DOCX 14 kb) [file 10578_2020_1038_MOESM1_ESM.docx]

**Supplementary File 1: Search terms for MEDLINE**

Term 1: Alcohol use

(alcohol us* OR alcohol abus* OR alcohol drink* OR alcohol drinking OR alcohol consum* OR binge drink* OR heavy drink* OR moderate drinking OR drinking behavior* OR Drinking problem* OR ethanol OR ethanol consum* OR ethanol abus* OR ethanol us* OR alcoholism OR alcoholic OR alcoholic beverage* OR alcohol exposure OR ethanol exposure OR alcohol dependen* OR ethanol dependen* OR AUDIT OR CAGE).mp

Term 2: Household

(Household OR family OR family environment OR family histor* OR parent* OR mother OR father OR carer* OR primary caregiver OR caregiver OR caregiver* OR relative$ OR cohabitant$ OR grandmother OR grandfather).mp

Term 3: Young Person

(Child* OR Children OR young children OR young child OR young boy* OR young girl* OR youngster* OR childhood OR early childhood OR late childhood OR infant* OR toddler OR adolescent$ OR youth* OR teen OR teenager* OR preadolescence OR pre-adolescence OR preadolescent OR pre-adolescent OR boy* OR girl* OR son* OR daughter* OR minor* OR juvenile* OR newborn OR schoolboy* OR schoolgirl* OR offspring* OR kindergarten).mp

Term 4: Neurodevelopmental outcome(s)

(development* OR child development* delay* OR childhood disability OR child disability IQ OR intelligence quotient OR intelligence test* OR learning disabilit* OR learning disorder* OR developmental disability* OR psychological OR psychological disorder* OR developmental OR developmental disorder* OR communication OR communicat* disorder* OR articulation disorder* OR Neurodevelopmental disorder* OR language delay* OR communication disorder* OR communication development* disorder OR communicat* dysfunction* OR behavior problem$ OR behavior difficulties OR Acquired language disability OR speech delay* OR language delay* OR speech disorder* OR language disorder* OR mental health OR social behavior$ OR abnormal behavior$ OR under-nutrition OR neurodevelopment* OR cognitive problem* OR cognitive delay* OR cognitive development OR delinquen* OR suicid* OR truan* OR disobedien* OR destructive OR teen pregnancy OR expulsion OR Conduct disorder* OR Oppositional defiant disorder* OR Disruptive behaviour* OR Disruptive behaviour* OR Disruptive behavior disorder* OR disruptive behaviour disorder* OR absenteeism OR lesson disrupt* OR class disrupt* OR risk* sexual behavior* OR risk* sexual behaviour* OR school failure OR drop-out OR absenteeism OR pregnancy).mp
